# Human PBMCs Form Lipid Droplets in Response to Spike Proteins

**DOI:** 10.3390/microorganisms11112683

**Published:** 2023-11-01

**Authors:** Kokilavani Sivaraman, Paco Pino, Guillaume Raussin, Stephanie Anchisi, Charles Metayer, Nicolas Dagany, Julia Held, Sabine Wrenger, Tobias Welte, Maria J. Wurm, Florian M. Wurm, Beata Olejnicka, Sabina Janciauskiene

**Affiliations:** 1 Department of Respiratory Medicine, German Center for Lung Research (DZL), Biomedical Research in Endstage and Obstructive Lung Disease Hannover (BREATH), Hannover Medical School, 30625 Hannover, Germany; 2 ExcellGene SA, 1970 Monthey, Switzerland; 3 Faculty of Life Sciences, École Polytechnique Fédérale de Lausanne, 1015 Lausanne, Switzerland

**Keywords:** recombinant spike protein, human PBMCs, lung microvascular endothelial cells, cytokines, chemokines, lipid droplets, lipid metabolism genes

## Abstract

Intracellular lipid droplets (LDs) can accumulate in response to inflammation, metabolic stresses, and other physiological/pathological processes. Herein, we investigated whether spike proteins of SARS-CoV-2 induce LDs in human peripheral blood mononuclear cells (PBMCs) and in pulmonary microvascular endothelial cells (HPMECs). PBMCs or HPMECs were incubated alone or with endotoxin-free recombinant variants of trimeric spike glycoproteins (Alpha, Beta, Delta, and Omicron, 12 µg/mL). Afterward, cells were stained with Oil Red O for LDs, cytokine release was determined through ELISA, and the gene expression was analyzed through real-time PCR using TaqMan assays. Our data show that spikes induce LDs in PBMCs but not in HPMECs. In line with this, in PBMCs, spike proteins lower the expression of genes involving lipid metabolism and LD formation, such as SREBF1, HMGCS1, LDLR, and CD36. On the other hand, PBMCs exposed to spikes for 6 or 18 h did not increase in IL-1β, IL-6, IL-8, MCP-1, and TNFα release or expression as compared to non-treated controls. Thus, spike-induced LD formation in PBMCs seems to not be related to cell inflammatory activation. Further detailed studies are warranted to investigate in which specific immune cells spikes induce LDs, and what are the pathophysiological mechanisms and consequences of this induction in vivo.

## 1. Introduction

Lipid droplets (LDs) are cytoplasmic organelles coated by phospholipids and structural proteins; perilipins, covering a hydrophobic core composed of triacylglycerol and cholesteryl esters; and a variable content of proteins [1]. The accumulation of LDs within non-adipocyte cells, such as leukocytes, epithelial and endothelial cells, hepatocytes, and cancer, has been reported. According to current knowledge, LDs are not only fat-storing intracellular structures but also players in cell signaling, lipid metabolism, membrane trafficking, and the production of inflammatory mediators [2]. LDs have also been linked to protein storage, for instance, in temporal storage of unfolded membrane proteins before proteasomal degradation [3].

Lipid droplet biogenesis is a regulated process that involves various cellular and molecular mechanisms, like increased lipid uptake, de novo lipid synthesis, and lipolysis. Nevertheless, mechanisms involved in LD accumulation following pathogen infection are still not completely understood. Various pathogen-associated molecular patterns can induce LDs by involving Toll-like receptor (TLR) signaling [4]. For example, lipopolysaccharide (LPS) can trigger LD formation in macrophages [5] and endothelial cells [6] via TLR signaling and a following increase in glucose uptake and glycolysis, leading to increased lipid biosynthesis and accumulation of LDs. The virally driven induction of LDs has been shown to be dependent on epidermal growth factor receptor signaling [7]. Recent studies demonstrate that type II pneumocytes and monocytes from COVID-19 patients are characterized by the pronounced accumulation of LDs [8], and that in multiple cell lines, severe acute respiratory syndrome coronavirus 2 (SARS-CoV-2) induces LD accumulation to benefit viral replication [9,10]. However, how SARS-CoV-2 triggers LD accumulation remains unknown.

SARS-CoV-2 virus contains four structural proteins, namely the spike, membrane, envelope, and nucleocapsid proteins. The trimer spike (S) glycoprotein is a sole SARS-CoV-2 viral membrane protein that binds to the angiotensin-converting enzyme 2 receptor on the target cell and mediates virus–cell fusion [11,12]. Typically, virions are decorated with 25–50 spike trimers, although others can contain more than 90 trimers [13], which are very immunogenic [14]. As spike is the most immunogenic of coronaviruses proteins, it is a major focus for vaccine, therapeutic, and diagnostic development. A new study reported that the spike protein interferes with metabolic and autophagic pathways in host cells [15,16], and thus might affect LD formation. Herein, we aimed to investigate whether recombinant trimeric spike glycoproteins can induce LDs in human total peripheral blood mononuclear cells (PBMCs) and/or in lung microvascular endothelial cells (HPMECs).

## 2. Materials and Methods

### 2.1. Spike Proteins

The widely spread SARS-CoV-2 variants were designated as Alpha (B.1.1.7), Beta (B.1.351), Gamma (P.1), Delta (B.1.617.2), and Omicron (B.1.1.529). We employed highly purified and endotoxin-free trimeric SARS-CoV-2 spike variants engineered for a high-yield production by ExcellGene (Monthey, Switzerland) to be used for research and development of novel vaccines [17]. Secreted forms of trimeric spike protein variants were produced in CHO cells based on the CHO-vector pXLG-6 by ExcellGene SA as described previously [18]. LAL (limulus amebocyte lysate) assay was used to detect bacterial endotoxins in proteins (Pierce™ chromogenes Endotoxin Quant Kit, Thermo Fisher Scientific, Rockford, IL, USA). For cell treatment, we used a constant spike concentration (12 µg/mL) selected for previous experiments with the “Wuhan” variant of the SARS-CoV-2 spike [19].

### 2.2. PBMC Isolation, Treatments, and Cytokine/Chemokine Analyses

PBMCs were isolated from fresh peripheral human donor blood by using Lymphosep (PL-15-M, c.c.pro, Oberdorla, Germany) discontinuous gradient centrifugation according to the protocol of the manufacturer. Isolated total PBMCs were suspended in serum-free RPMI Medium 1640 (Gibco/Thermo Fisher Scientific, Paisley, UK), and were incubated for 6 or 18 h alone or with spikes (12 µg/mL each) in multiwell plates with cell-repellent surface (Greiner Bio-One GmbH, Frickenhausen, Germany) at 37 °C with 5% CO_2_. Cell supernatants were used for ELISA-based quantitative analysis of IL-1β (DY201, sensitivity: 3.91–250 pg/mL), TNF-α (DY210, sensitivity: 15.6–1000 pg/mL), IL-6 (DY206, sensitivity: 9.38–600 pg/mL), and CCL2/MCP-1 (DY279, Sensitivity: 15.6–1000 pg/mL). ELISA kits were purchased from R&D Systems (Minneapolis, MI, USA). Microplate reader Tecan Infinite M200 was used (Männedorf, Switzerland).

### 2.3. Primary Human Pulmonary Microvascular Endothelial Cell Culture

Human pulmonary microvascular endothelial cells (HPMECs; Promocell, Heidelberg, Germany) were cultured in MV-2 endothelial cell growth medium (Promocell, Heidelberg, Germany) at 37 °C with 5% CO_2_. HPMECs (passages 4–6, at 70–90% confluence) were incubated for 6 or 18 h alone or with spikes (12 µg/mL). Experiments were performed in serum-free cell growth medium.

### 2.4. Oil Red O Staining

Total PBMCs or HPMEC were incubated for determined time points on coverslips alone or with spikes (12 µg/mL) for 18 h. Afterward, cells were fixed with 3% paraformaldehyde and stained with Oil Red O (O-0625, Sigma-Aldrich, Burlington, Massachusetts, USA) for LDs and with hematoxylin for the nuclei. The number of cells positive for LDs was manually counted and the percentage was calculated based on the total number of cells per field (for each condition, 10 different fields were evaluated). Images from HPMECs were taken at 1000-fold magnification using a 100× oil immersion objective (Leica Microsystems, Wetzlar, Germany) on a Leica ICC 50 HD microscope. Images from PBMCs were taken at 1000-fold magnification using BX43 light microscope (Olympus Life Science Solutions, Tokyo, Japan) with a 100× oil immersion objective (Olympus, Tokyo, Japan).

### 2.5. RNA Isolation and Gene Expression Analysis through RT-PCR

Human PBMCs cultured for 6 h without or with addition on spikes (as above) were collected for gene expression analysis. Total RNA isolation, cDNA synthesis, and target gene analysis were performed as previously described [19]. The following primers were purchased from Thermo Fisher Scientific: CD36 (Hs00169627_m1), PLIN2 (Hs00605340_m1), DDIT3 (Hs00358796_g1), CXCL8 (Hs00174103_m1), IL1B (Hs01555410_m1), TNFA (Hs00174128_m1), CCL2 (Hs00234140_m1), IL6 (Hs00985639_m1), SREBF1 (Hs01088691_m1), LDLR (Hs01092524_m1), HMGCS1 (Hs00266810_m1). HPRT1 (Hs02800695_m1) was used as a housekeeping gene in the same run. The measured gene expression was calculated according to the 2∆Ct method (Ct value of target gene − Ct value of reference gene).

### 2.6. Statistical Analysis

Statistical analysis and graphical data presentation of ELISA and RT-PCR were performed by using GraphPad Prism (Version 9.1.2 (226), GraphPad Software). One-way ANOVA was applied to compare between groups. Data are presented as mean and standard deviation if the normality test did not fail. If the normality test failed, the nonparametric Kruskal–Wallis one-way analysis followed by Mann–Whitney rank-sum test was performed. A *p*-value below 0.05 was considered as significant.

## 3. Results

### 3.1. Spike Proteins Are Endotoxin-Free and Do Not Induce Cytokine/Chemokine Release and Expression in PBMCs

**Figure 1:**
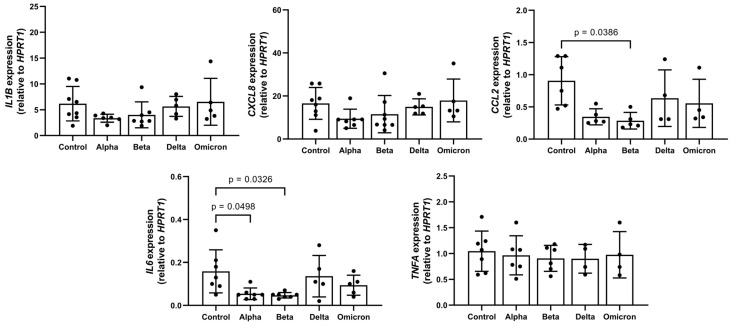
Effects of spike variants on cytokine/chemokine gene expression. Total PBMCs from healthy donors were cultured for 6 h in the presence of 12 µg/mL of spike variants or alone. RNA was isolated and analyzed through real-time qPCR. Data are shown as mean (SD) from N = 7 independent experiments. All analyses were carried out in duplicates. *p*-values were calculated using one-way ANOVA comparing each condition with “Control”. A *p*-value below 0.05 was considered as significant.

Previous studies reported that spike proteins contaminated with LPS are pro-inflammatory [20] and that LPS can induce LD formation [6]. According to an LAL assay, in all spike variants, endotoxin levels were below detection limits. In concordance with this, in supernatants of PBMCs treated with spikes, the levels of all analyzed pro-inflammatory cytokines/chemokines (IL-1β, TNF-α, IL-6, and CCL2/MCP-1) were below the detection limits of ELISA assays (Supplementary Table S1, Supplementary Figure S1). As illustrated in Figure 1, these spike variants had no significant effects on the expression of *IL1B*, *CXCL8*, and *TNFA*. The expression of *CCL2* was significantly down regulated by the “Beta” variant and that of *IL6* by “Alpha” and “Beta”. In a previous study using the “Wuhan” variant of the SARS-CoV-2 spike, LPS induced a strong increase in cytokine gene expression, confirming that the test works [19].

### 3.2. Spike Proteins Induce Lipid Droplet (LD) Formation in PBMCs

**Figure 2:**
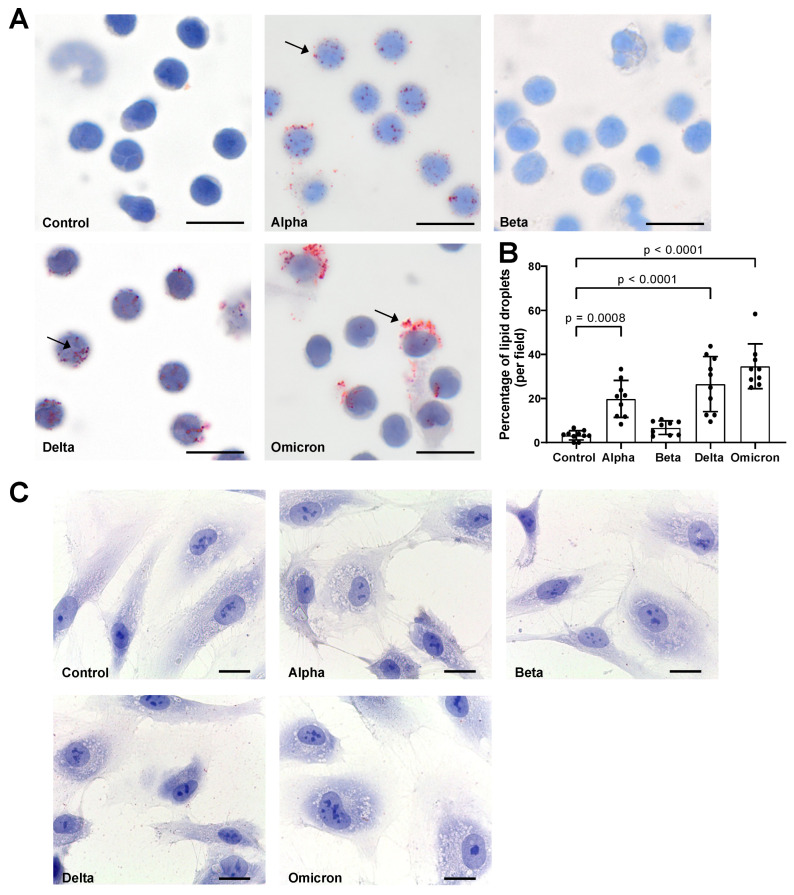
Effects of spike proteins on lipid droplet (LD) formation. (**A**) PBMCs from healthy donors were cultured for 18 h alone or in the presence of spike variants. Lipid droplets were stained with Oil red O and nuclei were stained with hematoxylin. Arrows indicate lipid droplets. Images were taken at 1000-fold magnification using 100× oil immersion objective (Olympus). Scale bars indicate 5 µm. One representative image is shown out of ten images taken from three independent experiments. (**B**) The number of cells positive for LDs was manually counted and the percentage was calculated based on the number of cells per field. Data are presented as mean (SD). *p*-values were calculated using one-way ANOVA comparing each condition with “Control”. A *p*-value below 0.05 was considered as significant. (**C**) HPMECs were cultured for 18 h alone or in the presence of spike variants. Lipid droplets were stained with Oil red O and nuclei were stained with hematoxylin. Images were taken at 1000-fold magnification using 100× oil immersion objective (Leica). Scale bars indicate 10 µm. One representative image is shown out of ten images taken from three independent experiments.

As shown in Figure 2A,B, spike variants added to total PBMCs for 18 h to varying degrees induced LD formation. Notably, spike “Beta” showed a lower effect on LDs as compared to other variants. Moreover, the spike-induced LD distribution and sizes differed. While “Alpha” and “Delta” variants induced smaller-size LDs in almost all cells, “Omicron”-induced LDs were larger and mainly occurred in large cells (Figure 2). In contrast, spikes in HPMECs did not induce LD formation (Figure 2C), and we did not perform further analyses with these cells.

**Figure 3:**
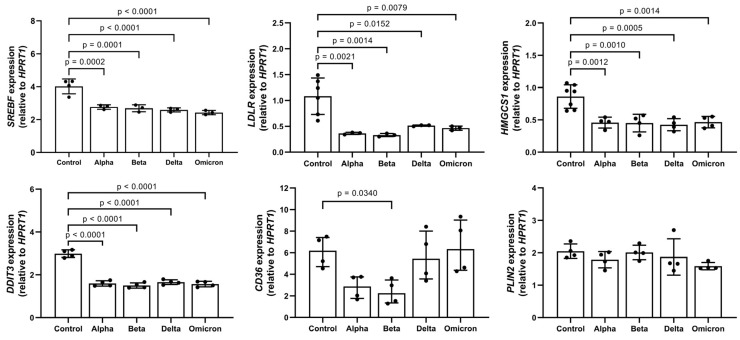
Impact of spike variants on lipid gene expression. PBMCs from healthy donors were cultured for 6 h alone or in the presence of spike proteins. RNA was isolated and analyzed using real-time qPCR. Data are shown as mean (SD) from N = 3 to N = 5 independent experiments. All analyses were carried out in duplicates. *p*-values were calculated using one-way ANOVA comparing each condition with “Control”. A *p*-value below 0.05 was considered as significant.

In parallel to LD formation, the expression of genes related to lipid metabolism, such as sterol regulatory element-binding transcription factor 1 (*SREBF1*), 3-Hydroxy-3-Methylglutaryl-CoA Synthase 1 (*HMGCS1*), low-density lipoprotein receptor (*LDLR*), and DNA damage-inducible transcript 3 (*DDIT3*), also known as C/EBP homologous protein (CHOP), significantly decreased in spike-treated PBMCs. Moreover, spikes to different degrees lowered the expression of fatty acid translocase, also known as (*CD36*), but did not affect perilipin 2 (*PLIN2*) expression (Figure 3).

## 4. Discussion

In this study, we show that endotoxin-free spike variants of SARS-CoV-2 added to human PBMCs within 18 h induce LD formation. The cytoplasmic accumulation of LDs in leukocytes and other cells can be a hallmark of inflammation [21,22,23,24]. Although the molecular mechanisms that govern LD biogenesis are incompletely understood, it is thought that inducible LD may occur due to inflammatory-stimuli-induced lipid uptake, lipolysis inhibition, and/or new lipid synthesis. For example, resting PBMCs show no LDs but can rapidly form LDs when activated through bacterial and/or host-generated cytokines and chemokines [24,25]. We therefore investigated the effects of spike proteins on cytokines/chemokine production in human total PBMCs. According to previous studies, the effects of spike proteins on inflammatory markers are sterility- and time-dependent, with the highest effect observed at 8 h post stimulation [20,26]. Therefore, we first confirmed that the spike proteins are endotoxin-free and decided to analyze cytokine/chemokine production in human PBMCs after 6 h of exposure to spikes. Under this experimental condition, none of the spikes increased pro-inflammatory cytokine/chemokine release or expression. Likewise, spikes did not induce IL-1β or IL-6 after 18 h. Hence, our data imply that LD formation in PBMCs is not solely dependent on the amplified cytokine/chemokine production. Of note, Yao et al. showed that resting primary monocytes harbor cytoplasmic angiotensin converting enzyme 2 (ACE-2) protein, which could be translocated to the cell surface upon TLR stimulation [27]. Therefore, TLR-activated monocytes express surface ACE-2, which allows the infection of cells by SARS-CoV-2 [27]. Although we did not examine the intracellular entry of the spike proteins, our data suggest that the TLR-induced pro-inflammatory signaling is not responsible for spike-induced LD formation. Whether spike proteins per se can induce ACE2 translocation to the immune cell surface by exploiting it for intracellular entry remains to be further investigated. In support for this latter idea, none of the spike variants induced LDs in primary human lung endothelial cells, which, according to recent findings, express very low levels of the SARS-CoV-2 receptor ACE2, and are therefore unlikely to be infected with SARS-CoV-2 [28]. On the other hand, LD formation in endothelial cells is a component of endothelial inflammation induced by LPS or TNF-α [6]. The absence of LDs in spike-treated HPMECs argues against the pro-inflammatory activation of endothelial cells by spike proteins and suggests a putative importance of ACE2 in spike-induced LD formation.

Intriguingly, in response to spikes, PBMCs formed LDs under complete lipid deprivation (serum-free medium) conditions. It is important to point out that the ACE2 receptor seems to localize with lipid rafts, allowing an interaction between spike and immune cells and the entry of the virus into the immune cell [29,30]. The relocation of ACE2 to the non-raft environment following cholesterol depletion could reduce the susceptibility to infections (in our case, an interaction with spikes) but cannot eliminate them [29]. According to various studies, the formation of LDs might be necessary for the protection of cell integrity and function during stress [31,32,33]. Hence, hypothetically, under nutrient-limited conditions, an interaction between the spike protein and cells might induce stress-related LD formation in the majority of PBMCs. Spike proteins induced LD formation but also lowered the expression of genes known to be involved in lipid metabolism, such as *HMGCS1*, *LDLR1*, and *SREBF1*. The *HMGCS1* is the rate-limiting enzyme of the cholesterol biosynthesis pathway, whereas *LDLR1* mediates cholesterol influx [34,35]. Hence, the spike-induced reduction in *HMGCS1* and *LDLR1* expression in PBMCs supports the notion that strong lipid deprivation may lead to LD formation. This might also be dependent on the reduction in expression of *SREBF1*, a key transcriptional factor that regulates the expression of *HMGCS1* and *LDLR*.

PLIN2 is the second Plin-family member encoding an LD surface-coating protein that is constitutively expressed in essentially all cells [36,37]. Pancreatic beta cells have been reported to increase the PLIN2 transcript and protein upon exposure to stress-inducing substances [38]. However, in monocytic cells, PLIN2 appears to be primarily regulated at the protein level. For example, in macrophages, the snake venom toxin B induced PLIN2 recruitment to LDs [39]. Pisano et al. showed that monocytic PLIN2 protein levels were significantly higher in children with overweight/obesity compared to normal-weight controls, whereas *PLN2* mRNA levels showed no significant difference [40]. Likewise, spike proteins did not affect the expression of the *PLIN2* gene in PBMCs. Most cells have high *PLIN2* mRNA but contain a modest amount of protein because of its rapid proteasomal degradation that is not associated with LDs [41]. This may explain why spikes showed no effect on *PLIN2* transcript levels.

As compared to controls, PBMCs treated with spikes showed a slight reduction in expression of the *CD36* transcript. CD36 is involved in the regulation of fatty acid storage or usage [42], and it is known as a fatty acid transporter protein, which belongs to the class B scavenger receptor family. Some studies show that the uptake of fatty acids is required for CD36-dependent LD formation [43]. Since our experiments were performed under serum-free conditions, LD formation was probably independent of CD36 expression.

Finally, the reduction in the expression of genes related to lipid metabolism and LD formation in PBMCs cultured with spikes under lipid starvation may simply be associated with a blockade of lipid release caused by cellular stress. This property might be very important for immune cells exposed to rapidly changing conditions of stress-like feeding/starvation or hypoxia/reoxygenation [44,45]. In line with this, a decreased *DDIT3* expression in human PBMCs exposed to spikes might be related to anti-damage and/or cell survival mechanisms [46].

Data from our study and other experimental models imply that spikes interfere with lipid metabolism pathways [15,16,47], and this warrants further investigations.

## 5. Conclusions

LDs are considered to originate from ER, and their number and size may vary depending on cell type and stimuli. Our results show that under lipid-free culture conditions, spike proteins of SARS-CoV-2 without triggering inflammatory cytokines/chemokines induce LD deposition in human PBMCs. Whether this spike-induced LD formation is beneficial or detrimental remains to be investigated.

## Data Availability

All data generated or analyzed during this study are included in this published article.

## Supplementary Materials

The following supporting information can be downloaded at https://www.mdpi.com/article/10.3390/microorganisms11112683/s1, Figure S1: Standard curves of the ELISAs, Table S1: ELISA OD values.
