# Supplementary material for: Human PBMCs Form Lipid Droplets in Response to Spike Proteins
Source: Microorganisms. 2023 Nov 1;11(11):2683. doi: 10.3390/microorganisms11112683 (PMC10672762; doi:10.3390/microorganisms11112683)
Supplement: Supplementary file 1 [file microorganisms-11-02683-s001.zip › microorganisms-2545051-supplementary.pdf]

Supplemantary Table S1: ELISA OD values: Difference data (OD_{450nm} - OD_{540nm})

ELISA	Donor	Control	Alpha	Beta	Delta	Omicron
IL1-β	1	0.0848	0.0786	0.0754	0.0770	0.0851
		0.0798	0.0769	0.0753	0.0761	0.0829
	2	0.0738	0.0852	0.0834	0.0745	0.0707
		0.0724	0.0782	0.0816	0.0765	0.0711
	3	0.0674	0.0945	0.0722	0.0938	0.1301
		0.0677	0.0946	0.0722	0.0910	0.1271
	4	0.0848	0.0786	0.0754	0.0770	0.0851
		0.0798	0.0769	0.0753	0.0761	0.0829
IL-6	1	0.0674	0.0945	0.0722	0.0938	0.1301
		0.0677	0.0946	0.0722	0.0910	0.1271
	2	0.0921	0.0867	0.0725	0.0831	0.0941
		0.0863	0.0872	0.0736	0.0820	0.0947
	3	0.0640	0.0733	0.1059	0.1015	0.0916
		0.0665	0.0691	0.1001	0.0965	0.0898
	4	0.0402	0.0331	0.0335	0.0341	0.0414
		0.0406	0.0336	0.0328	0.0341	0.0421
MCP-1	1	0.0480	0.1787	0.0325	0.0621	0.0967
		0.0484	0.1738	0.0312	0.0576	0.0923
	2	0.0999	0.0339	0.0325	0.0279	0.0346
		0.0944	0.0335	0.0324	0.0280	0.0352
	3	0.0366	0.0299	0.0318	0.0317	0.0322
		0.0323	0.0286	0.0314	0.0311	0.0303
	4	0.0342	0.0208	0.0221	0.0202	0.0217
		0.0324	0.0206	0.0214	0.0192	0.0209
TNF-α	1	0.0347	0.0335	0.0339	0.0336	0.0390
		0.0326	0.0327	0.0331	0.0324	0.0388
	2	0.0317	0.0339	0.0343	0.0343	0.0337
		0.0316	0.0338	0.0347	0.0336	0.0315
	3	0.0521	0.0631	0.0689	0.0658	0.0684
		0.0518	0.0653	0.0682	0.0640	0.0668
	4	0.0499	0.0516	0.0529	0.0512	0.0550
		0.0500	0.0525	0.0541	0.0518	0.0567

Difference data (OD at measurement wavelength 450 nm minus OD at reference wavelength 540nm) are given. The values are around or below the low standard of IL1-β, IL-6, MCP-1, and TNF-α ELISAs (see supplementary figure 1).

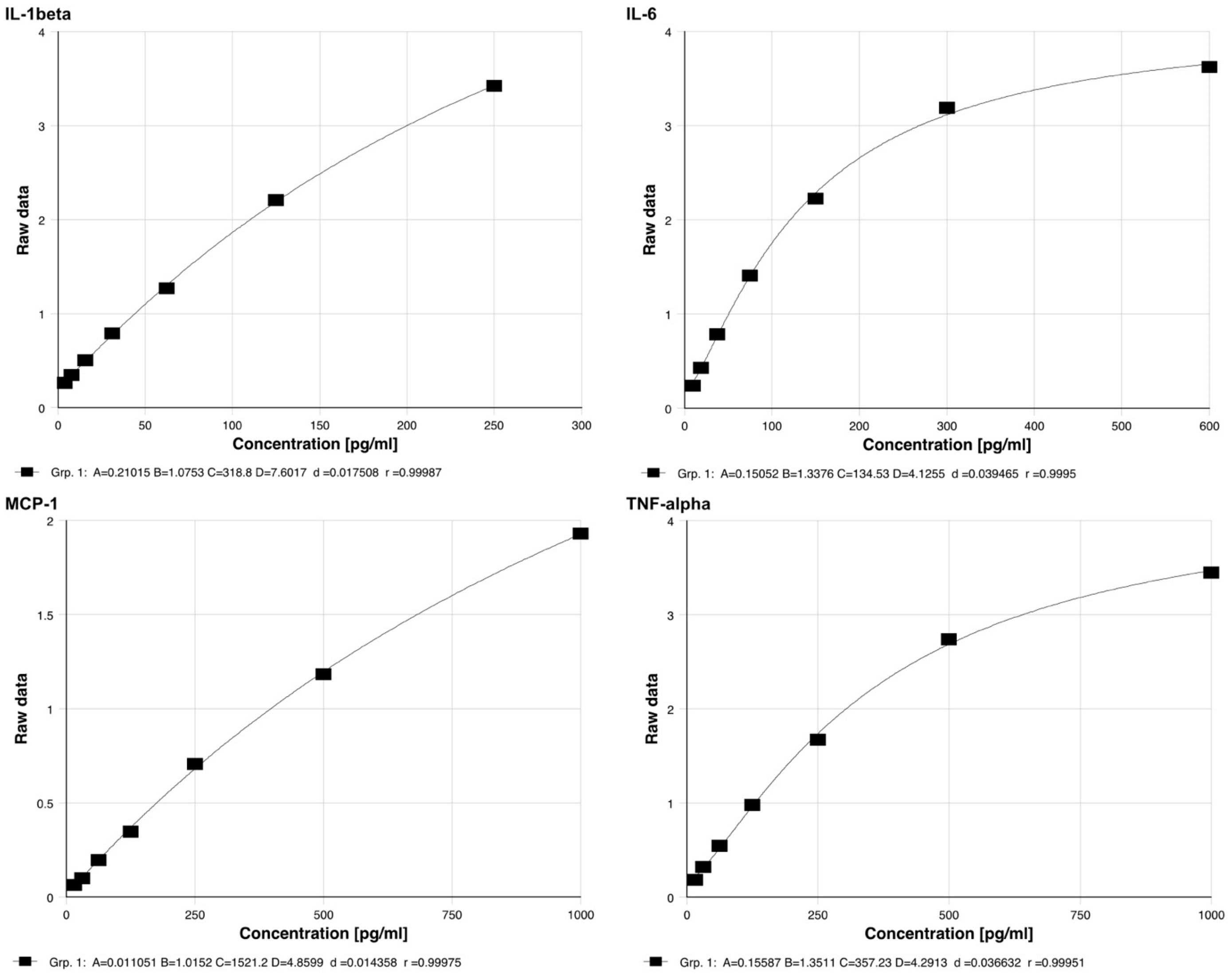

Supplementary Figure S1: Standard curves of the used ELISAs. Plotted are OD difference data (OD_{450nm} - OD_{540nm}) against the concentration (pg/ml).
